# A hybrid RNA FISH immunofluorescence protocol on *Drosophila* polytene chromosomes

**DOI:** 10.1186/s13104-023-06482-0

**Published:** 2023-09-07

**Authors:** Hannah E. Gilbonio, Gwyn L. Puckett, Erica Nguyen, Leila E. Rieder

**Affiliations:** 1 Department of Biology, Emory University, Atlanta, GA USA; 2 Piedmont Virginia Community College, Charlottesville, VA USA; 3 Perelman School of Medicine, University of Pennsylvania, Philadelphia, PA USA

**Keywords:** RNA FISH, IF, *Drosophila*, Polytene chromosomes, Transcription factor, Histone genes, Mxc

## Abstract

**Objectives:**

Investigating protein-DNA interactions is imperative to understanding fundamental concepts such as cell growth, differentiation, and cell development in many systems. Sequencing techniques such as ChIP-seq can yield genome-wide DNA binding profiles of transcription factors; however this assay can be expensive, time-consuming, may not be informative for repetitive regions of the genome, and depend heavily upon antibody suitability. Combining DNA fluorescence in situ hybridization (FISH) with immunofluorescence (IF) is a quicker and inexpensive approach which has historically been used to investigate protein-DNA interactions in individual nuclei. However, these assays are sometimes incompatible due to the required denaturation step in DNA FISH that can alter protein epitopes, hindering primary antibody binding. Additionally, combining DNA FISH with IF may be challenging for less experienced trainees. Our goal was to develop an alternative technique to investigate protein-DNA interactions by combining RNA FISH with IF.

**Results:**

We developed a hybrid RNA FISH-IF protocol for use on *Drosophila melanogaster* polytene chromosome spreads in order to visualize colocalization of proteins and DNA loci. We demonstrate that this assay is sensitive enough to determine if our protein of interest, Multi sex combs (Mxc), localizes to single-copy target transgenes carrying histone genes. Overall, this study provides an alternative, accessible method for investigating protein-DNA interactions at the single gene level in *Drosophila melanogaster* polytene chromosomes.

**Supplementary Information:**

The online version contains supplementary material available at 10.1186/s13104-023-06482-0.

## Introduction

The relationships between a DNA locus and the proteins that target that locus affect fundamental processes such as DNA replication, transcription, and repair [1]. A common technique used to investigate protein-DNA localization is ChIP-seq, which captures positional information of proteins across the genome. However, ChIP-seq involves several caveats: it is expensive, it requires access to sequencing platforms [2], and it is difficult to perform by inexperienced users. As an alternative to ChIP-seq, investigators utilize microscopy to reveal protein localization [3], monitor biochemical interactions between proteins and DNA [4], and quantify binding mechanisms that lead to the formation of protein-DNA complexes [5]. To confirm whether a protein of interest is targeting a specific genomic locus, investigators have historically combined DNA fluorescent in situ hybridization (DNA FISH) with protein immunofluorescence (IF) [6, 7]. However, the protocol for DNA FISH is often incompatible with protein immunohistochemistry; it involves a DNA denaturation step that can reverse chemical crosslinks and denature protein epitopes, thus hindering primary antibody binding [8–10].

Combining DNA FISH with IF on *Drosophila* polytene chromosomes is a historically invaluable method for cytological analysis. Polytene chromosomes are formed through repetitive cycles of DNA endoreduplication without nuclear division. These polyploid cells can contain up to 1024 copies of the genome [11]. This amplification of the genome gives rise to defined chromosomal banding patterns that represent chromatin regions and allows investigators to analyze relatively high-resolution protein binding patterns [12, 13]. Although researchers have applied DNA FISH and IF on polytenes in the past [6], the aforementioned incompatibility presents a need for an alternative method to investigate protein-DNA interactions. To address this need, we developed a hybrid RNA FISH-IF method that indirectly marks genes on *Drosophila* polytene chromosomes. Unlike DNA FISH, RNA FISH identifies the RNA transcripts that surround its parent gene [14], thus providing indirect genomic locus information. As RNA is single-stranded, RNA FISH does not require a denaturation step [15], which renders it more compatible with immunohistochemistry and more accessible for less-experienced trainees than DNA FISH.

We tested the ability of RNA FISH probes to mark genomic loci on polytene chromosomes, determined if single molecule RNA (smRNA) probes hybridize to local RNA or DNA, and optimized our hybrid RNA FISH-IF protocol. We used the *D. melanogaster* histone gene array as our model system. In the wildtype *D. melanogaster* genome*,* the endogenous histone locus consists of five histone genes arranged in a single histone array (HA). Arrays are tandemly repeated ~ 107 times at a single locus [16]. We also used engineered fly lines that carry transgenes with varying numbers of HAs, where even a single HA can attract specific transcription factors [17, 18]. Notably, this gene array titration allowed us to incrementally optimize the sensitivity of our RNA FISH-IF hybrid assay towards single-gene detection. We verified expected protein-DNA interactions on polytenes marked with histone smRNA FISH probes by immunostaining with Multi sex combs (Mxc), a protein that only targets HAs (including both endogenous and transgenic HAs) [19–21]. We sought to expand this technique for those working with any *Drosophila* transgene marked by reporters, specifically by leveraging smRNA probes targeting common markers like *mini-white*. However, we found that most single-copy genes did not give strong signal by smRNA FISH, suggesting that transcriptional level in salivary gland tissue contributes to detection limits. Overall, we present a protocol for investigating and visualizing protein binding at specific genomic locations that is inexpensive, quick, and accessible to *Drosophila* investigators at all levels.

## Methods

### Fly stocks

*Drosophila melanogaster* fly lines were maintained on standard cornmeal-molasses food at 18°C. Third instar larvae were used for dissections. Fly stocks were obtained from Bloomington *Drosophila* Stock Center (*y,w*: stock #1495) or as gifts from the Duronio and Marzluff laboratories [17, 22].

### Antibodies and RNA FISH probes

The following antibodies were used for immunostaining: guinea pig anti-Mxc (1:5000; gift from Duronio and Marzluff laboratories); Goat anti-Guinea Pig AlexaFluor 488 (1:1000) (Invitrogen #A-11073); Goat anti-Guinea Pig AlexaFluor 647 (1:1000) (Invitrogen #A-21450). Custom RNA FISH probe sets were obtained from LGC Biosearch Technologies using the Stellaris Probe Designer version 4.2. All RNA FISH probes used in this procedure were coupled to Quasar 570 or Quasar 670 fluorophores.

### RNase decontamination

RNase-free reagents were made from DEPC-treated water and RNase-free 1X PBS [23]. RNase AWAY (Spectrum Chemical MFG Corp #970-66898) was used to clean all surfaces and tools and used filtered pipette tips. Slide holders were cleaned by washing them in a mixture of DEPC-treated water and RNase AWAY.

### Sample preparation

Salivary glands were dissected from third instar larvae and fixed with three separate fixatives (Fix 1: 4% paraformaldehyde, 1% Triton X-100 in RNase-free 1X PBS) (Fix 2: 4% paraformaldehyde, 50% acetic acid in DEPC-treated water) (Fix 3: 1:2:3 lactic acid:DEPC-treated water:glacial acetic acid) [18] using DEPC-treated reagents and RNase-free materials. Salivary glands were transferred to the 1:2:3 fixative (~ 20 µL) on a siliconized (RainEX) 22 mm square coverslip surface. A glass slide was placed on the coverslip containing the salivary glands and third fix and quickly flipped over. Slides were frozen in liquid nitrogen and immediately removed their coverslips. Slides were briefly stored in RNase-free 1X PBS and immediately proceeded with immunohistochemistry.

### Immunohistochemistry

Slides were washed in 1% Triton X-100 in RNase-free 1X PBS and rocked gently for 10 min. Slides were washed twice for 5 min in RNase-free 1X PBS the sample perimeter of each slide was marked with an ImmEdge pen (Vector Laboratories #H-4000) in between both washes. 250 µL of blocking solution (0.5% UltraPure BSA (Invitrogen #AM2616) in RNase-free 1X PBS) was added to the sample area of each slide and the slides were incubated at room temperature in a dark humid chamber for 1 h, shaking gently. Using coverslips, slides were incubated with 40 µL of diluted primary antibody in blocking solution overnight in a dark, sealed, humid chamber at 4°C. Slides were washed 3 times for 5 min in RNase-free 1X PBS. 40ul of diluted secondary antibody in blocking solution was applied to each slide (including coverslips) and slides were incubated for 2 h in a dark humid chamber at room temperature. Slides were washed 3 times for 5 min in RNase-free 1X PBS. 250 µL of post-fixative (4% paraformaldehyde in RNase-free 1X PBS) was added to each sample area for 3 min at room temperature. Each slide was then washed 3 times for 5 min in RNase-free 1X PBS.

### RNA FISH

Slides were washed for 5 min in ~ 30 mL Wash Buffer (1:10:1 20 × SSC:DEPC-treated water:deionized formamide) at room temperature. 125 µL of Hybridization Buffer (100 mg/mL dextran sulfate and 10% formamide in 2 × SSC and DEPC-treated water) and diluted RNA FISH probe (3 µL of 25 µM probe:120 µL Hybridization Buffer) was added to slides (including coverslips) which were incubated in a dark, humid, sealed chamber at 37°C overnight (~ 16 h). Slides were washed twice for 10 min in prewarmed (37°C) Wash Buffer in the dark. 250 µL Wash Buffer and diluted DAPI (25 ng/mL DAPI) was added onto the sample area and incubated slides in a dark, humid chamber at 37°C for 30 min. Slides were mounted with ~ 15 µL of VECTASHIELD Antifade Mounting Medium (VWR #101098–042) and No. 1.5 coverslips. Slides were sealed with nail polish. Slides were immediately imaged, as we found this gave the strongest FISH signal.

### Microscopy

A widefield Zeiss AXIO Scope A1 microscope with X-Cite 120 LED Boost fluorescent light source and a 40 × Plan-neofluar 0.75 NA objective paired with ZEN 3.6 (blue edition) was used for imaging. Zen (.czi) files were visualized with ImageJ Version 1.53t.

### RNase treatment

After dissecting salivary glands, glands were treated with 0.1% Triton X-100 for 2 min prior to adding 100 µg/mL RNase A (NEB #T3018L) and incubated for 1 h at room temperature [24] before fixation.

## Results

**Fig. 1:**
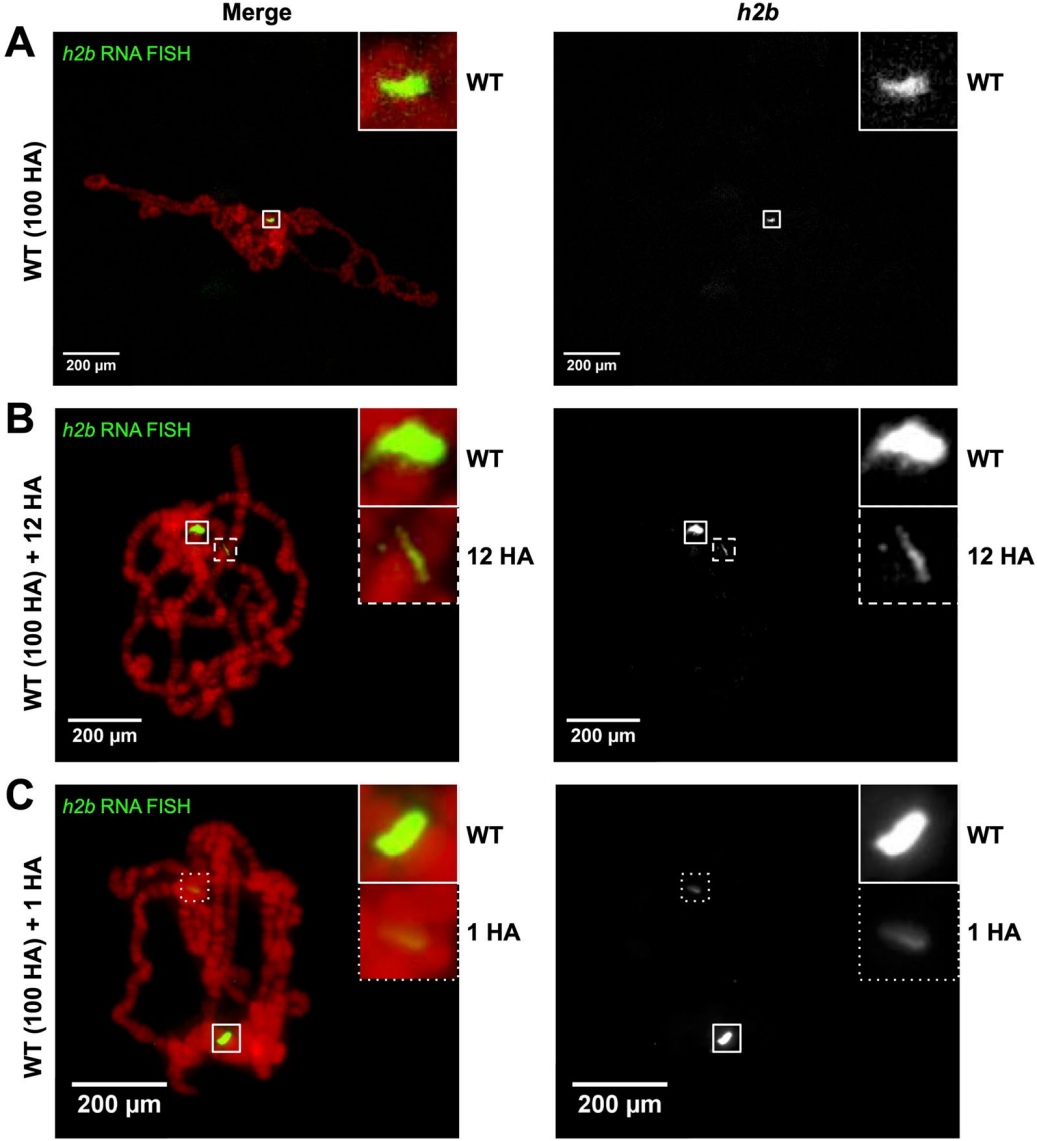
*Histone2b* (*h2b*) RNA FISH (green) on **A** wildtype (WT; 100 histone arrays), **B** wildtype with a 12 copy histone array transgene (WT + 12 HA), and **C** wildtype with a single copy histone array transgene (WT + 1 HA) on *D. melanogaster* polytene chromosomes. DNA is stained with DAPI (red)

A high concentration of transcripts surrounds the parent gene locus in many cells. We therefore hypothesized that RNA FISH would mark genetic loci on *Drosophila* polytene chromosomes. We performed RNA FISH on wild-type polytene chromosomes using smRNA FISH probe sets against either *histone2b* (*h2b*) or *histone3* (*h3*) transcripts because they are the longest histone genes. The endogenous histone locus, which carries ~ 100 tandem histone gene arrays [16], is located on chromosome 2L near the centromere. Our *h2b* and *h3* RNA FISH probe sets effectively targeted this region (Fig. 1A, Additional file 1: Fig. S1A). Since most genes do not exist in multiple copies, we next performed the same RNA FISH assay on transgenic lines carrying HA transgenes, either with 12 tandem copies of the histone gene array or a single array. Our *h2b* and *h3* probe sets effectively detected these transgenes (Fig. 1B–C**, **Additional file 1: Fig. S1B–C).

**Fig. 2:**
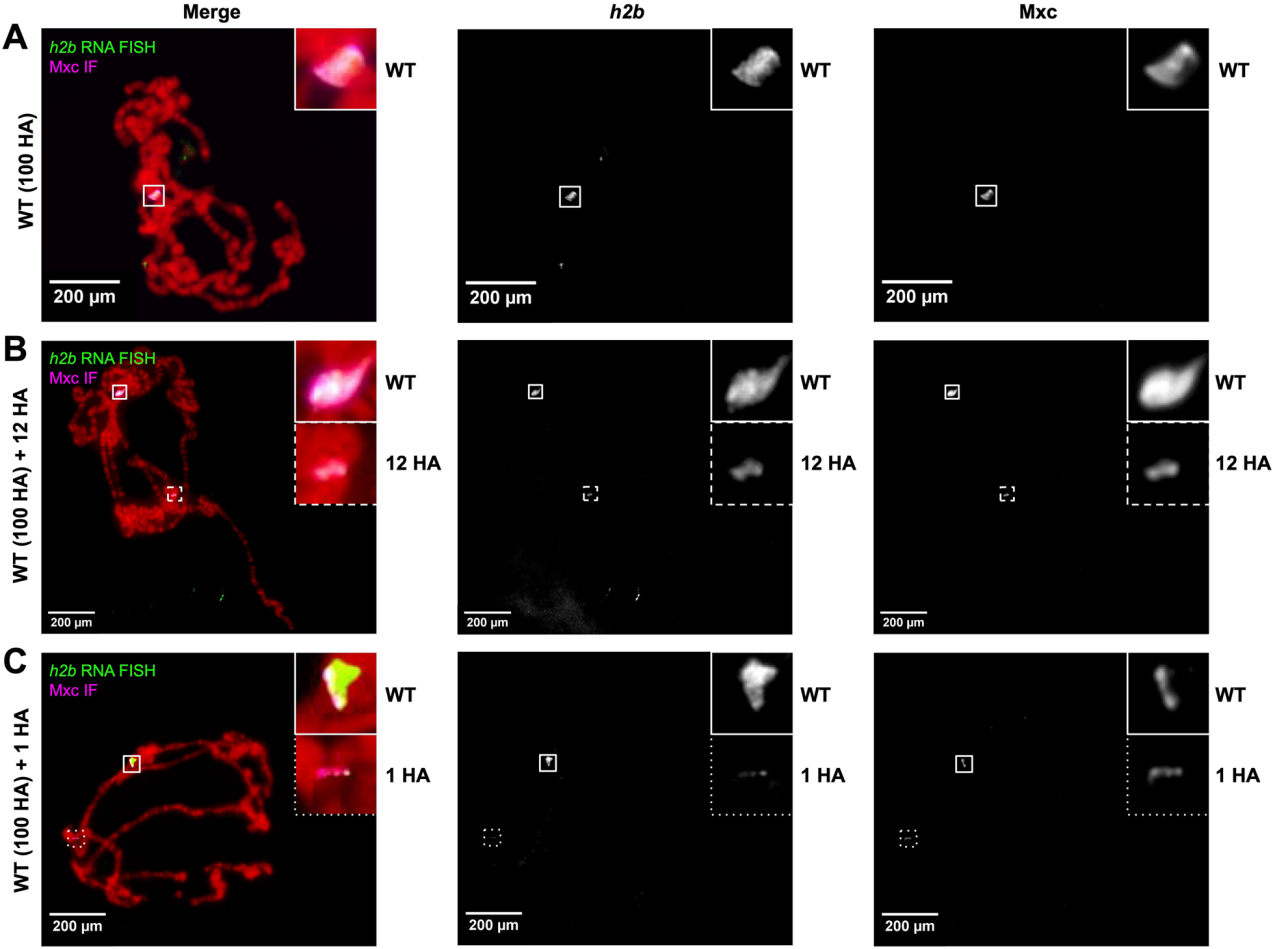
*Histone2b* (*h2b*) RNA FISH (green) and Mxc IF (magenta) on **A** wildtype (WT; 100 histone arrays), **B** wildtype with a 12 copy histone array transgene (WT + 12 HA), and **C** wildtype with a single copy histone array transgene (WT + 1 HA) on *D. melanogaster* polytene chromosomes. DNA is stained with DAPI (red)

To confirm that our RNA FISH probe sets are detecting histone loci, and to confirm that our RNA FISH protocol is compatible with IF, we combined our RNA FISH assay with antibody detection of the histone-locus specific factor Multi sex combs (Mxc) [22, 25]. Mxc also localizes to transgenes carrying histone gene arrays on polytene chromosomes [17, 18, 22]. We observed that Mxc signal colocalizes with *h2b* and *h3* RNA FISH, confirming these locations as the endogenous histone locus (Fig. 2A, Additional file 1: Fig. S2A) and the transgenic loci (Fig. 2B–C**, **Additional file 1: Fig. S2B–C). While developing this protocol, we intentionally conducted IF first because certain RNA FISH regents can alter protein epitopes. We included a postfixative step in between IF and RNA FISH to preserve the IF signal [23], included larger-volume washes, and increased the concentration of probe to 3 µL of 25 µM probe per 100 µL hybridization buffer (see Methods). All of these steps contributed to boosting RNA FISH signals when combined with IF.

**Fig. 3:**
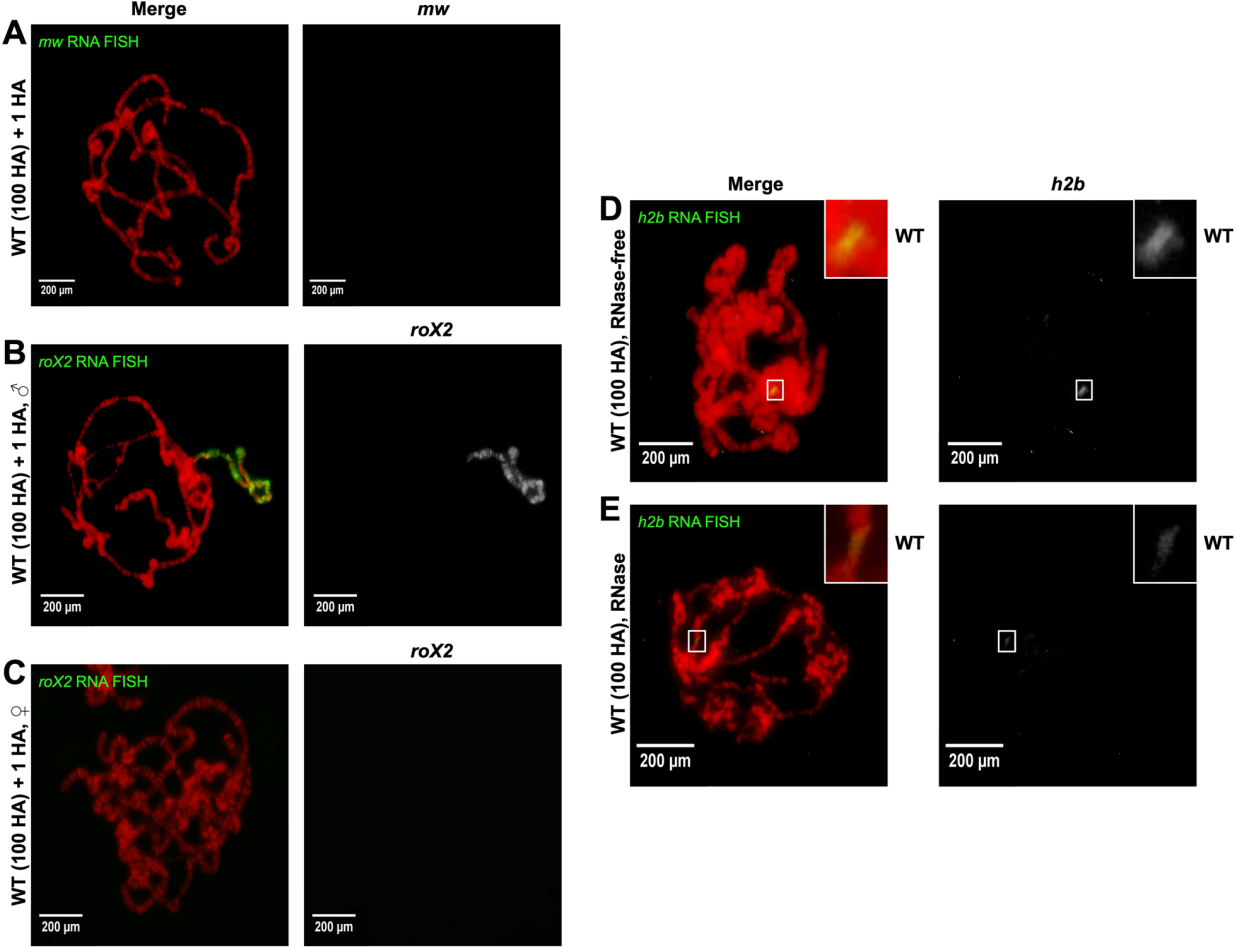
RNA FISH on wildtype *D. melanogaster* polytene chromosomes with a single copy histone array transgene (WT + 1 HA) using probes against **A** *mini-white* (*mw*; green)*,* **B** *roX2* (green) in males, and **C** *roX2* (green) in females. *Histone2b* (*h2b*) RNA FISH (green) on wildtype polytenes (WT; 100 histone arrays) **D** without RNase treatment and **E** with RNase treatment. DNA is stained with DAPI (red)

Given that we verified Mxc colocalization with histone genes, we wanted to further test the ability of both assays to mark different single-copy genomic loci on polytenes. We tested our procedure on *mini-white*, a common transgene marker that we use to mark HA transgenes on chromosome 3R, as well as *roX2,* an X-linked long non-coding RNA that is only expressed in males. *RoX2* participates in *Drosophila* dosage compensation and coats the male X-chromosome [26, 27]. We did not detect a signal for the *mini-white* gene (Fig. 3A). While we clearly detected *roX2* on male X-chromosomes (Fig. 3B), as previously documented, we saw no RNA FISH signals for the *roX2* locus in female polytenes (Fig. 3C). To verify that our RNA FISH probes sets were binding to mRNA transcripts and not binding directly to DNA, we introduced RNase A into the procedure. We observed a large reduction in signal (Fig. 3D–E), suggesting that our RNA FISH probes are binding directly to local mRNA.

## Discussion

Our goal in developing a hybrid RNA FISH and IF protocol for polytene chromosomes was to create an alternative method to visualize protein-DNA localization in *Drosophila*. Here, we successfully combined RNA FISH and IF to visualize Mxc (a protein that only targets histone genes) colocalizing with histone gene loci. This proof of principle suggests that our hybrid assay could be applied to other proteins and genomic loci of interest. Unfortunately, we found that *mini-white*, a common transgene marker in *Drosophila*, was not visible by RNA FISH on polytene chromosomes. Considering that the wild-type *white* gene has very low expression levels in salivary glands [28], we are not surprised by the absence of *mini-white* signal. These observations suggest that our RNA FISH technique is more applicable for visualizing loci of genes that are highly expressed in larval salivary glands. Due to the relatively low cost of the protocol and reagents in addition to the 3 day turnaround time, we believe this hybrid RNA FISH and IF procedure is an accessible method for testing protein-DNA colocalization, especially for those with limited wet lab experience.

## Limitations

This protocol is most likely limited to investigating genes in *Drosophila* polytene chromosomes that have high expression in *Drosophila* larval salivary glands. We only tested this method in *Drosophila melanogaster*, yet we see no reason why this assay cannot be applied to other *Drosophila* species. Polytene chromosomes represent up to 1024 copies of the genome (11); we did not test our protocol on diploid cells. However, as others have observed that RNA FISH signals often cluster around genomic loci, RNA FISH-IF is likely a viable technique to simultaneously visualize DNA loci and proteins, even in diploid cells. Although RNA FISH may be used for quantitative analyses, here we use it solely as a locational marker.

### Supplementary Information

**Additional file 1: Figure S1.** *Histone3* (*h3*) RNA FISH (green) on **A** wild-type (WT; 100 histone arrays), **B** wild-type with a 12 copy histone array transgene (WT + 12 HA), and **C** wild-type with a single copy histone array transgene (WT + 1 HA) on *D. melanogaster* polytene chromosomes. DNA is stained with DAPI (red). **Figure S2.** *Histone3* (*h3*) RNA FISH (green) and Mxc IF (magenta) on **A** wild-type (WT; 100 histone arrays), **B** wild-type with a 12 copy histone array transgene (WT + 12 HA), and **C** wild-type with a single copy histone array transgene (WT + 1 HA) on *D. melanogaster* polytene chromosomes. DNA is stained with DAPI (red).

## Data Availability

Data are available from the corresponding author upon request. The authors will supply a detailed protocol upon request.

## Abbreviations

smRNA: Single molecule RNA
FISH: Fluorescent in situ hybridization
IF: Immunofluorescence
Mxc: Multi sex combs
ChIP-seq: Chromatin immunoprecipitation sequencing
HA: Histone array
DEPC: Diethyl pyrocarbonate
