# Supplementary material for: A hybrid RNA FISH immunofluorescence protocol on Drosophila polytene chromosomes
Source: BMC Res Notes. 2023 Sep 7;16:197. doi: 10.1186/s13104-023-06482-0 (PMC10486132; doi:10.1186/s13104-023-06482-0)
Supplement: Supplementary file 1 — Additional file 1: Figure S1. Histone3 (h3) RNA FISH (green) on A wild-type (WT; 100 histone arrays), B wild-type with a 12 copy histone array transgene (WT + 12 HA), and C wild-type with a single copy histone array transgene (WT + 1 HA) on D. melanogaster polytene chromosomes. DNA is stained with DAPI (red). Figure S2. Histone3 (h3) RNA FISH (green) and Mxc IF (magenta) on A wild-type (WT; 100 histone arrays), B wild-type with a 12 copy histone array transgene (WT + 12 HA), and C wild-type with a single copy histone array transgene (WT + 1 HA) on D. melanogaster polytene chromosomes. DNA is stained with DAPI (red). [file 13104_2023_6482_MOESM1_ESM.pdf]

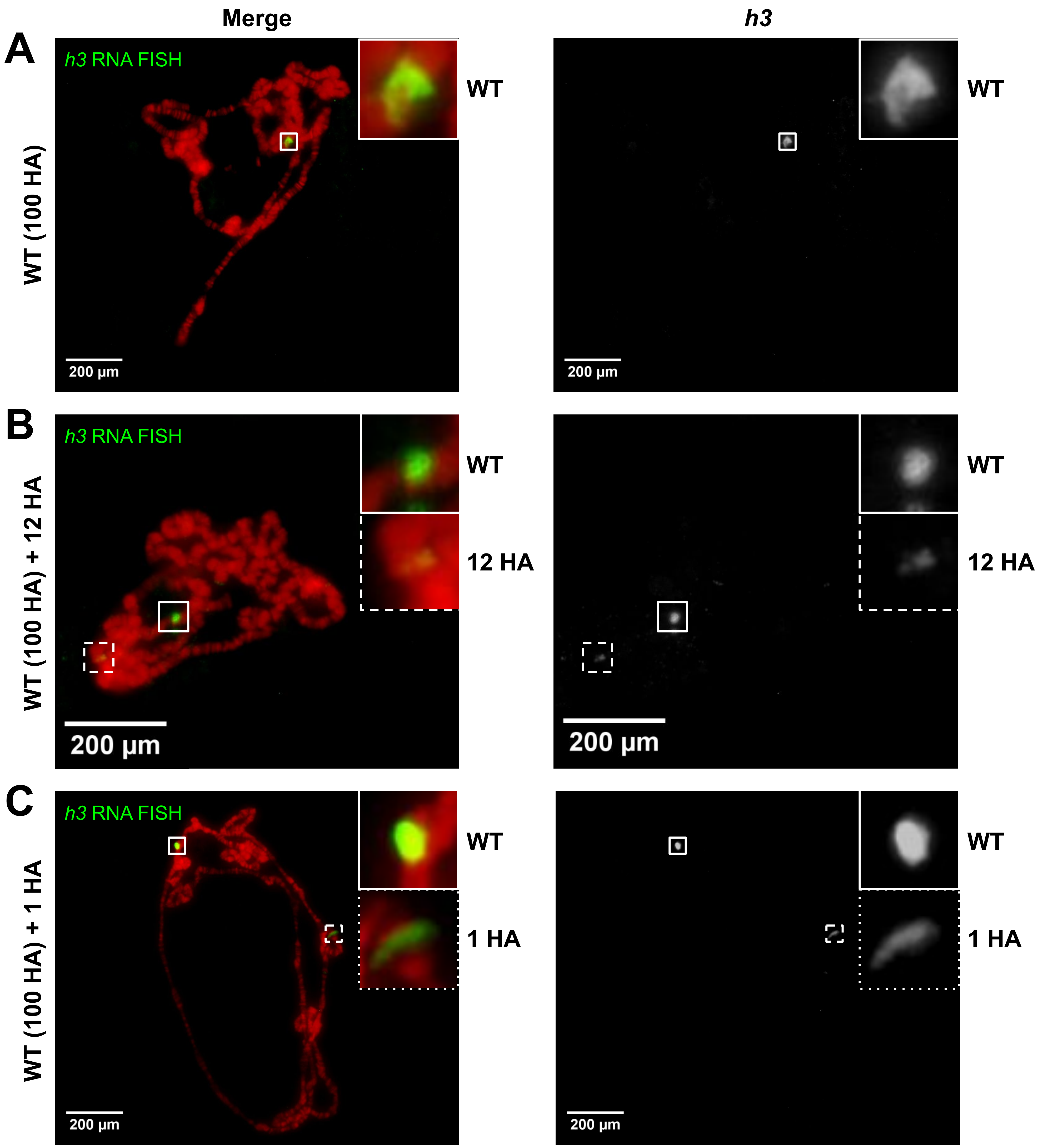

Figure S1: *H3* RNA FISH (green) on **A** wild-type (WT; 100 histone arrays), **B** wild-type with a 12 copy histone array transgene (WT + 12 HA), and **C** wild-type with a single copy histone array transgene (WT + 1 HA) on *D. melanogaster* polytene chromosomes. DNA is stained with DAPI (red)

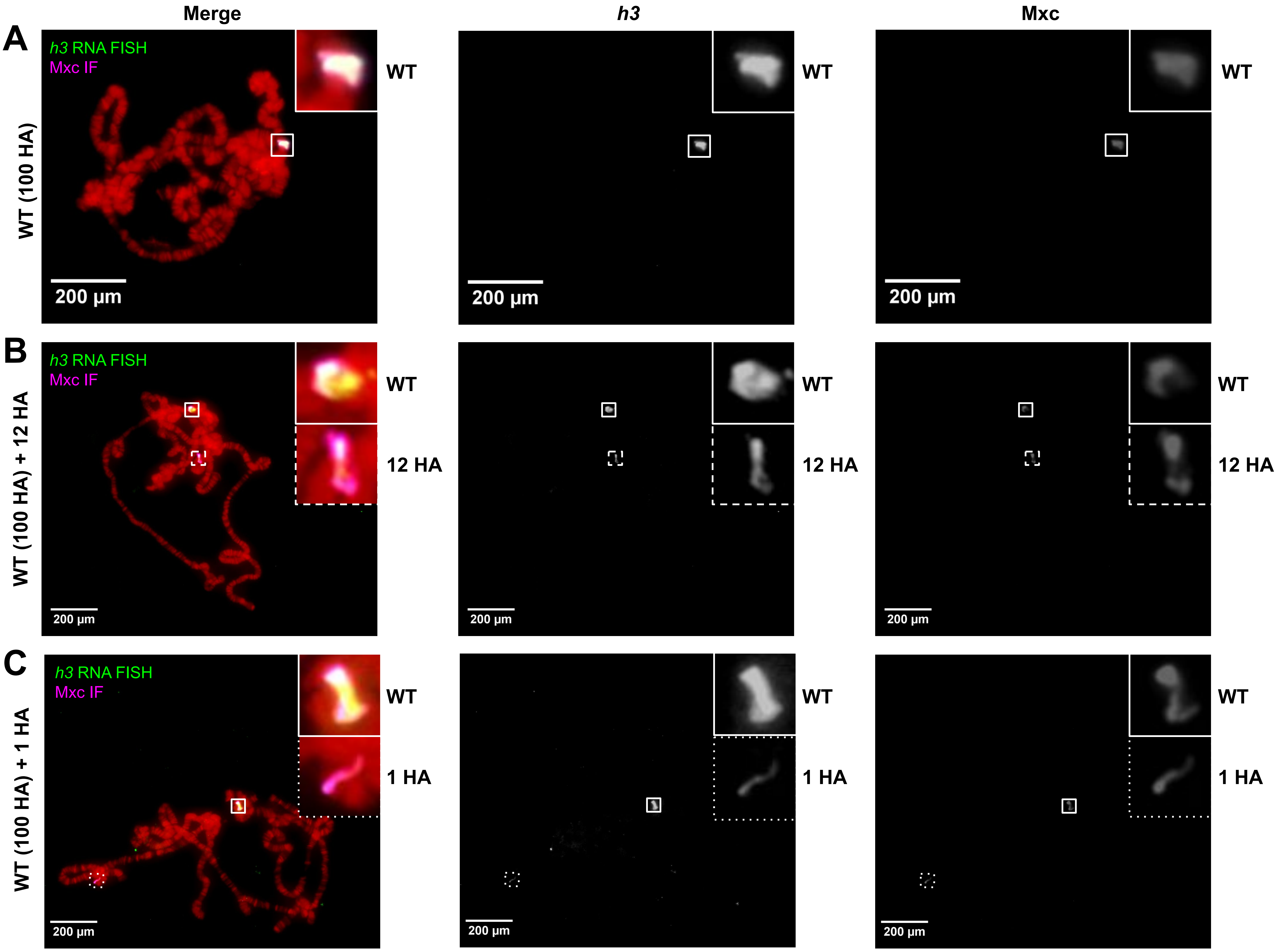

Figure S2: *H3* RNA FISH (green) and Mxc IF (magenta) on **A** wild-type (WT; 100 histone arrays), **B** wild-type with a 12 copy histone array transgene (WT + 12HA), and **C** wild-type with a single copy histone array transgene (WT + 1HA) on *D. melanogaster* polytene chromosomes. DNA is stained with DAPI (red).
